# Supplementary material for: MetaRibo-Seq measures translation in microbiomes
Source: Nat Commun. 2020 Jun 29;11:3268. doi: 10.1038/s41467-020-17081-z (PMC7324362; doi:10.1038/s41467-020-17081-z)
Supplement: Supplementary file 10 — Supplementary Data 7 [file 41467_2020_17081_MOESM10_ESM.zip › File2/Confidence_High_Taxonomy/159447_out.krona.html]

Javascript must be enabled to view this page.

members
magnitude
magnitudeUnassigned
count
unassigned
taxon
rank

159447\_out

6


SRS097920\_contig\_number\_contig-100\_15247.136238
1

superkingdom
2
5

1239
phylum
4

4
class
186801

186802
order
4

3
541000
family

3
species

SRS018836\_contig\_number\_22621SRS143895\_contig\_number\_32604SRS148196\_contig\_number\_22041
1898205

1950825

SRS015578\_contig\_number\_26593
species
1

1
phylum
201174

class
1760
1

85011
order
1

2062
family
1

1
genus
1883

285578
species

SRS019445\_contig\_number\_contig-100\_8983.100017
1
